# Complete Mitochondrial DNA Analysis of Eastern Eurasian Haplogroups Rarely Found in Populations of Northern Asia and Eastern Europe

**DOI:** 10.1371/journal.pone.0032179

**Published:** 2012-02-21

**Authors:** Miroslava Derenko, Boris Malyarchuk, Galina Denisova, Maria Perkova, Urszula Rogalla, Tomasz Grzybowski, Elza Khusnutdinova, Irina Dambueva, Ilia Zakharov

**Affiliations:** 1 Institute of Biological Problems of the North, Russian Academy of Sciences, Magadan, Russia; 2 The Nicolaus Copernicus University, Ludwik Rydygier Collegium Medicum, Institute of Forensic Medicine, Department of Molecular and Forensic Genetics, Bydgoszcz, Poland; 3 Institute of Biochemistry and Genetics, Ufa Research Center, Russian Academy of Sciences, Ufa, Russia; 4 Institute of General and Experimental Biology, Russian Academy of Sciences, Ulan-Ude, Russia; 5 Vavilov Institute of General Genetics, Russian Academy of Sciences, Moscow, Russia; University of Cambridge, United Kingdom

## Abstract

With the aim of uncovering all of the most basal variation in the northern Asian mitochondrial DNA (mtDNA) haplogroups, we have analyzed mtDNA control region and coding region sequence variation in 98 Altaian Kazakhs from southern Siberia and 149 Barghuts from Inner Mongolia, China. Both populations exhibit the prevalence of eastern Eurasian lineages accounting for 91.9% in Barghuts and 60.2% in Altaian Kazakhs. The strong affinity of Altaian Kazakhs and populations of northern and central Asia has been revealed, reflecting both influences of central Asian inhabitants and essential genetic interaction with the Altai region indigenous populations. Statistical analyses data demonstrate a close positioning of all Mongolic-speaking populations (Mongolians, Buryats, Khamnigans, Kalmyks as well as Barghuts studied here) and Turkic-speaking Sojots, thus suggesting their origin from a common maternal ancestral gene pool. In order to achieve a thorough coverage of DNA lineages revealed in the northern Asian matrilineal gene pool, we have completely sequenced the mtDNA of 55 samples representing haplogroups R11b, B4, B5, F2, M9, M10, M11, M13, N9a and R9c1, which were pinpointed from a massive collection (over 5000 individuals) of northern and eastern Asian, as well as European control region mtDNA sequences. Applying the newly updated mtDNA tree to the previously reported northern Asian and eastern Asian mtDNA data sets has resolved the status of the poorly classified mtDNA types and allowed us to obtain the coalescence age estimates of the nodes of interest using different calibrated rates. Our findings confirm our previous conclusion that northern Asian maternal gene pool consists of predominantly post-LGM components of eastern Asian ancestry, though some genetic lineages may have a pre-LGM/LGM origin.

## Introduction

The territories of northern Asia are of crucial importance for the study of early human dispersal and the peopling of the Americas. Recent findings about the peopling of northern Asia reconstructed by archaeologists suggest that anatomically modern humans colonized the southern part of Siberia around 40 thousand years ago (kya) and the far northern parts of Siberia and ancient Beringia, a prerequisite for colonization of the Americas, by approximately 30 kya [1], [2]. Current molecular genetic evidence suggests that the initial founders of the Americas emerged from an ancestral population of less than 5,000 individuals that evolved in isolation, likely in Beringia, from where they dispersed southward after approximately 17 kya [3]–[13].

Despite the northern Asian populations are still underrepresented in the published complete genome mtDNA data sets, our knowledge of the fine-detailed mitochondrial DNA tree of northern Asians has been considerably improved recently, mainly due to the elaborate analyses of certain mtDNA haplogroups which are the most common in populations of northern Asia and America [4], [5], [7], [8], [10], [12]. Recently we have analyzed a large set of complete mtDNAs belonging to the most frequent haplogroups A, C and D as well as to some western Eurasian haplogroups found in northern Asian populations [8], [12]. As a result, it has been shown that majority of haplogroups C and D subclusters demonstrate the pre-LGM origin and expansion in eastern Asia, whereas the most of the southern and northeastern Siberian variants started to expand after the LGM. The Late Glacial re-expansion of microblade-making populations from the refugial zones in southern Yenisei and Transbaikal region of southern Siberia that started approximately 18 kya has been suggested as a major demographic process signaled in the current distribution of northern Asian-specific subclades of mtDNA haplogroups C and D. It has been shown also that both of these haplogroups were involved in migrations, from eastern Asia and southern Siberia to eastern and northeastern Europe, likely during the middle Holocene [12].

As far as uncovering all of the most basal variation in the northern Asian mtDNA haplogroups require major sampling and sequencing efforts with focusing on as much as possible diverse set of Siberian aboriginal populations we have further sampled two aboriginal populations from two different geographic regions of the northern and eastern Asia – Altaian Kazakhs from southern Siberia and Barghuts from Inner Mongolia, China and completely sequenced and analyzed an essential number of mtDNAs representing the rare and poorly characterized eastern Eurasian haplogroups which were revealed so far in northern Asia. We have paid a special attention to the 55 samples representing haplogroups B (n = 23), F2 (n = 1), M9 (n = 9), M10 (n = 5), M11 (n = 3), M13 (n = 2), N9a (n = 10), R9c1 (n = 1) and R11 (n = 1). Applying the newly updated mtDNA tree to the previously reported northern Asian and eastern Asian mtDNA data sets has resolved the status of the poorly classified mtDNA haplotypes and allowed us to obtain the coalescence age estimates of the nodes of interest using different calibrated rates.

## Results and Discussion

### MtDNA haplogroup profiles

Detailed sequence variations and haplogroup assignments of 149 Barghut and 98 Altaian Kazakh mtDNAs are presented in Table S1. A total of 36 haplogroups were observed in our samples, all within the three principal non-African macrohaplogroups: M, N and R. Table 1 presents the haplogroup frequencies of two populations studied. The eastern Eurasian component is represented by haplogroups A, N9a, and Y1, which belong to the major haplogroup N; by haplogroups B, F and R9c, which belong to macrohaplogroup R; and by different branches of macrohaplogroup M, such as C, D, G, M7, M9a, M13, and Z haplogroups. Both populations exhibit the prevalence of eastern Eurasian lineages accounting for 91.9% in Barghuts and 60.2% in Altaian Kazakhs. As in other populations of northern and eastern Asia [8], [12] haplogroups C and D are the most common in Barghuts and Altaian Kazakhs studied, accounting together for 55.7% and 34.7% of lineages, respectively. As can be expected, haplogroup G2 lineages, which occur with the highest frequencies in Mongolic-speaking populations [8] are more frequent in our Mongolic-speaking Barghut samples - 8.7%, as compared with 1% in Turkic-speaking Altaian Kazakhs (P = 0.01, Fisher's exact test). Meanwhile, Altaian Kazakhs exhibited a diverse set of the western Eurasian mtDNAs belonging to haplogroups H (13.3%), J (5.1%), HV (3.1%), U (10.2%), T (4%), R2 (1%) and I (3.1%), accounting together for 39.8% of lineages, whereas Barghuts demonstrate a lower contribution of this component (8.1%), represented only by haplogroups H (2%), HV (1.3%) and U (4.7%).

**Table 1:** mtDNA haplogroup frequencies in Barghuts and Altaian Kazakhs.

Hg	Barghuts(n = 149)		Altaian Kazakhs(n = 98)	
	n	%	n	%
A4	8	5.4	3	3.1
A8	1	0.7	0	0.0
B4	9	6.0	1	1.0
B5	3	2.0	3	3.1
C4	24	16.1	8	8.2
C5	5	3.4	0	0.0
C6	1	0.7	0	0.0
D2	3	2.0	0	0.0
D3	2	1.3	0	0.0
D4	47	31.5	22	22.4
D5	1	0.7	4	4.1
F1	4	2.7	5	5.1
F2	1	0.7	0	0.0
G2	13	8.7	1	1.0
G3	1	0.7	1	1.0
M13	2	1.3	0	0.0
M7	3	2.0	5	5.1
M9a	1	0.7	1	1.0
N9a	2	1.3	2	2.0
R9c1	1	0.7	0	0.0
Y1	1	0.7	1	1.0
Z*	4	2.7	2	2.0
H	3	2.0	13	13.3
J	0	0.0	5	5.1
HV	2	1.3	3	3.1
U2	1	0.7	1	1.0
U3	0	0.0	1	1.0
U4	0	0.0	4	4.1
U5	1	0.7	2	2.0
U7	1	0.7	0	0.0
U8	2	1.3	0	0.0
K	2	1.3	2	2.0
T*	0	0.0	2	2.0
T1	0	0.0	2	2.0
R2	0	0.0	1	1.0
I	0	0.0	3	3.1

### Population summary statistics, PC analysis and MDS plot

Internal population diversity indices and results of Tajima's D and Fu's Fs neutrality tests are presented in Table 2. Both studied populations exhibited high and similar diversity levels, as well as significant negative values for both Tajima's D and Fu's neutrality tests, suggesting past population expansion.

**Table 2:** Diversity indices and neutrality tests for the studied populations based on HVS1 variability data.

Population	n^a^	H (SE)^b^	K (K/n)^c^	S^d^	Pi (SE)^e^	θ_k_ (95% CI)	Tajima's D^f^	Fu's F_S_ f
Barghuts	149	0.988 (0.003)	97 (65)	90	6.143 (2.938)	119.43 (85.28–168.16)	−1.96	−24.98
Altaian Kazakhs	98	0.987 (0.003)	58 (59)	77	6.634 (3.16)	58.84 (39.29–88.42)	−1.81	−25.02

a Sample size.

b Sequence diversity (H) and standard error (SE).

c Number of different haplotypes and percentage of sample size in parentheses.

d Number of segregating sites.

e Average number of pairwise differences (Pi) with standard error (SE).

f All P values are <0.05 (for Tajima's D) and <0.02 for Fu's F_S_), except where noted.

**Figure 1:**
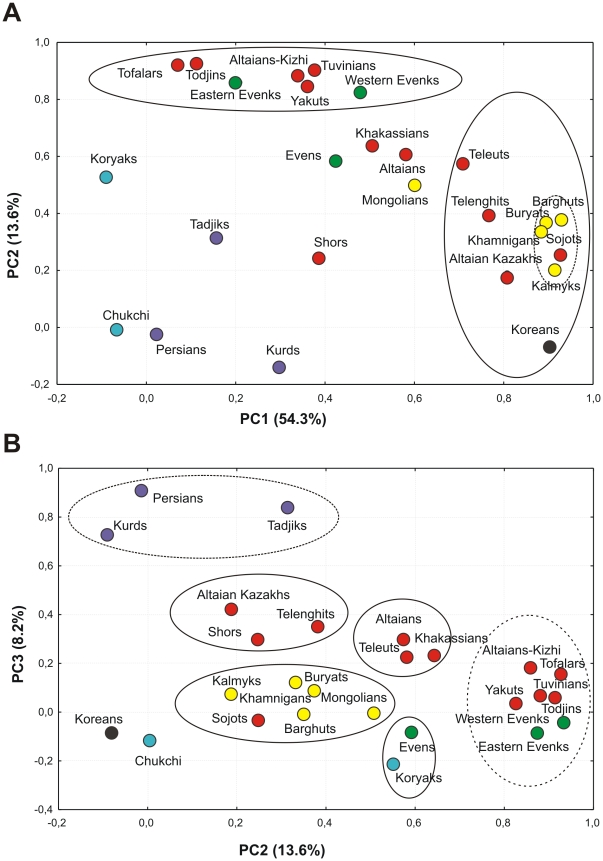
PC plots (A – PC1 vs PC2; B – PC2 vs PC3) based on mtDNA haplogroup frequencies for population samples from northern, eastern, central and western Asia. Linguistic affiliation of populations is indicated by different colors: Turkic group of Altaic family – in red, Mongolic group of Altaic family – in yellow, Tungusic group of Altaic family – in green, Northern group of Chukotko-Kamchatkan family – in blue, Indo-Iranian group of Indo-European family – in purple, language isolate – in black.

The basal mtDNA haplogroup frequencies of two populations studied and the 24 populations of western (Persians, Kurds), central (Tadjiks), eastern (Mongolians, Koreans), northern Asia (Tofalars, Tuvinians, Todjins, eastern and western Evenks, Yakuts, Altaians, Altaians-Kizhi, Teleuts, Telenghits, Khakassians, Shors, Evens, Chukchi, Koryaks, Buryats, Sojots, Khamnigans) Asia and eastern Europe (Kalmyks) published previously [8] were used as input vectors to perform a PC analysis. Figure 1 shows the PC plots for the first three PCs, which account for 54.3%, 13.6% and 8.2% of the total variance, respectively. The first two PCs reveal two major groups of populations. The first one is comprised of populations of Buryats, Barghuts, Khamnigans, Kalmyks and Sojots forming a distinct subcluster as well as populations of Altaian Kazakhs, Teleuts, Telenghits and Koreans, whereas the second cluster is constituted by the populations of Tofalars, Todjins, Tuvinians, eastern and western Evenks, Altaians-Kizhi and Yakuts. The PC3 essentially displays the close genetic proximity of the Indo-European-speaking populations – Persians, Kurds and Tadjiks (Figure 1), who are clearly separated from the other populations studied.

**Figure 2:**
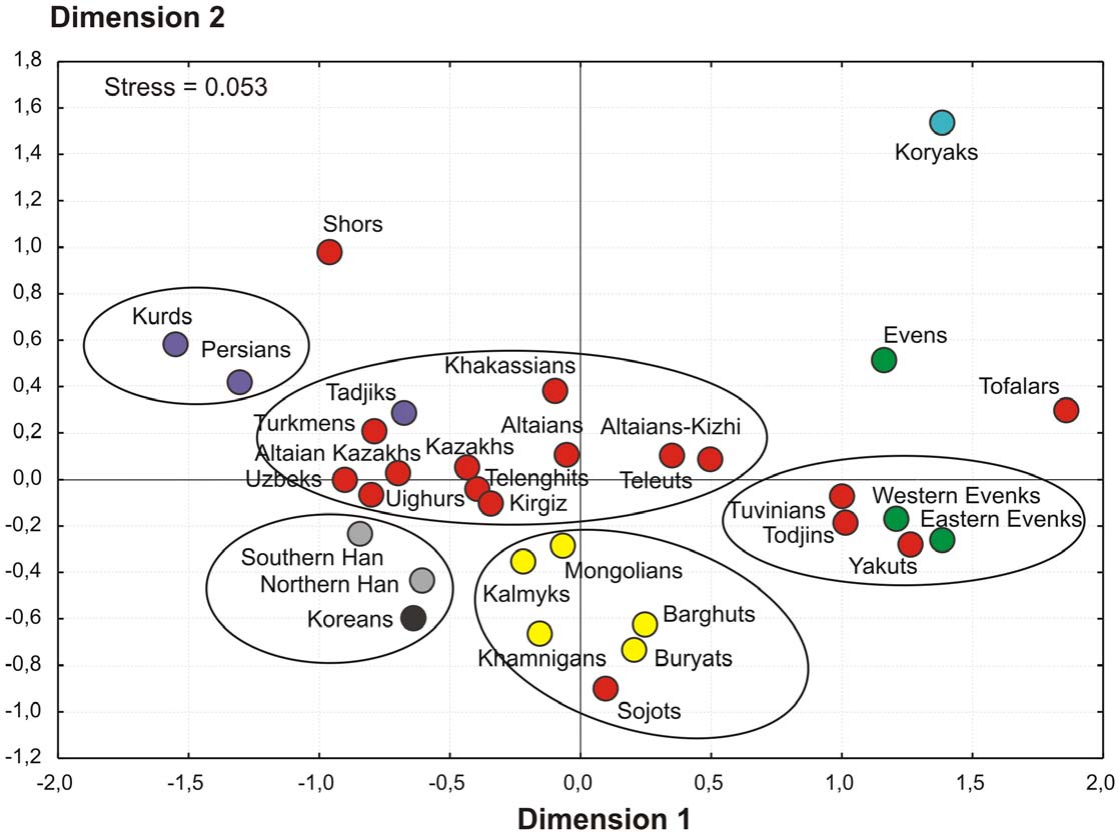
MDS plot based on FST statistics calculated from mtDNA HVS1 sequences for population samples from northern, eastern, central and western Asia. Linguistic affiliation of populations is indicated by different colors: Turkic group of Altaic family – in red, Mongolic group of Altaic family – in yellow, Tungusic group of Altaic family – in green, Northern group of Chukotko-Kamchatkan family – in blue, Indo-Iranian group of Indo-European family – in purple, Chinese group of Sino-Tibetan family – in grey, language isolate – in black.

The strong affinity of Altaian Kazakhs and populations of northern (Khakassians, Altaians, Altaians-Kizhi, Teleuts and Telenghits) and central (Tadjiks, Turkmens, Uzbeks, Uighurs, Kirghizs and Kazakhs) Asia is also evident from MDS analysis results (Figure 2), reflecting both strong influences of central Asian inhabitants on maternal diversity of Altaian Kazakhs as was previously reported [14] and essential genetic interaction between Altaian Kazakhs and the Altai region indigenous populations. Meanwhile, MDS plot as PC analysis previously reveals a close positioning of all Mongolic-speaking populations and Turkic-speaking Sojots related with them, thus suggesting their origin from a common maternal ancestral gene pool. The same trend is also evident for some of paternal lineages - a relatively high frequency of subhaplogroup C3d widespread in Mongolic-speaking populations was found in Sojots (53.6%), thus placing them closer to their Mongolic-speaking neighbors, than to other Turkic-speaking groups [15]. However, the Sojots are characterized by a relatively high frequency of the Y-chromosome haplogroup R1a1 (about 25%), which is typical for the Turkic-speaking populations such as Altaians, Teleuts and Shors, all characterized by the highest frequencies of R1a1 (about 50%) in Siberia [16]. Therefore, it seems that the Turkic males might have contributed genetically to the formation of Sojots, imposing a language of the Turkic group. In this scenario, most likely an elite dominance process should be assumed [17].

### Phylogeography of eastern Eurasian mtDNA haplogroups infrequent in populations of northern Eurasia

#### Haplogroups R11'B6 and B4'B5

Haplogroup B is found at relatively high frequencies in Mainland southeastern Asia (20.6%), Island southeastern Asia (15.5%), Oceania (10.2%), eastern Asia (10.5%) and America (24%), but occurs as rarely as 0.1–1% in the Volga-Ural region, the Caucasus, western and southern Asia. It is detected at a very low frequency in some populations of Europe. Haplogroup B is found at ∼3% overall in northern and central Asia, although it reaches >10% in a few Siberian populations (Table S2). Haplogroup B is identified by the presence of a 9-bp deletion in the COII/tRNALys intergenic region of mtDNA. Despite the 9-bp deletion has a high recurrence, it seems that together with transition 16189 it defines fairly well a monophyletic cluster, which consists of two subhaplogroups, B4 and B5. A sister clade of B4'B5, keeping the 16189 mutation and having additional polymorphism at np 12950, has been detected in eastern and Island southeastern Asia, being designated as R11'B6 [18], [19]. R11'B6 cluster is further subdivided in R11, lacking the 9-bp deletion, and B6, having this deletion. It is worthwhile to mention that R11 mtDNAs have been detected mainly in China, whereas B6 lineages are present both in eastern and Island southeastern Asia (Figure S1). Previous studies have proposed that haplogroup B4 arose ∼44 ka, most likely on the eastern Asian or southeastern Asian mainland, where it is dispersed especially around the coastal regions from Vietnam to Japan. It subdivided ∼35 ka into three main subclades: B4a, B4b'd, and B4c (with a subclade of B4b, B2, found exclusively in Native Americans and dated to ∼16 ka [5]). Subclades B4a and B4a1 are also likely to have arisen on the mainland, ∼24 ka and ∼20 ka, respectively, but B4a1a is restricted to offshore populations in Taiwan, Island southeastern Asia, and the Pacific [20]. Subclade B4a1a1a, defined by a transition at the control-region position 16247, also known as the Polynesian motif, is the most frequent subclade within B4a1a and approaches fixation in Polynesians. Based on complete mtDNA analysis data it has been shown that the motif most likely originated >6 ka in the close proximity of the Bismarck Archipelago, and its immediate ancestor is >8 ka old and virtually restricted to Near Oceania [20].

While there has been considerable recent progress in studying complete mitochondrial DNA variation of haplogroup B lineages in America [5], eastern [21] and southeastern Asia [22]–[25] and Oceania [20], [26] little comparable data is available for northern Asia. To date, only five haplogroup B complete mtDNA genomes from Siberian populations are known, which were sequenced and analyzed only with the aim of searching of the ancestors of Native American mtDNA haplogroups [27].

Here we present the reconstructed phylogeny of haplogroups R11'B6 and B4'B5 based on 247 complete mtDNA genomes including twenty three newly sequenced samples of haplogroup B from different populations of northern (Buryats, Khamnigans, Altaians-Kizhi, Yakut and Shor), eastern (Barghut) Asia and eastern Europe (Chuvashes from the Volga-Ural region) as well as one rare Altaian R11 sample. As can be seen from the phylogeny presented in Figure S1, the only Altaian R11 sample (Alt_158) and Han individual (QD8168) from Kong et al. [28] share transition at np 16390 and insertion of four cytosines at np 8278 and may therefore be ascribed to a new subclade R11b1 within R11b branch of haplogroup R11. Unfortunately, because of the small number of available R11b mtDNA genome sequences, we are unable to obtain unbiased age estimates for this subcluster, but taking into account the nearly exclusively Chinese distribution of R11 mtDNA lineages we may suppose that this specific Altaian R11b sequence points to a gene flow from China to southern Siberia, which might have occurred not earlier than 13–20 kya (Table S3).

**Figure 3:**
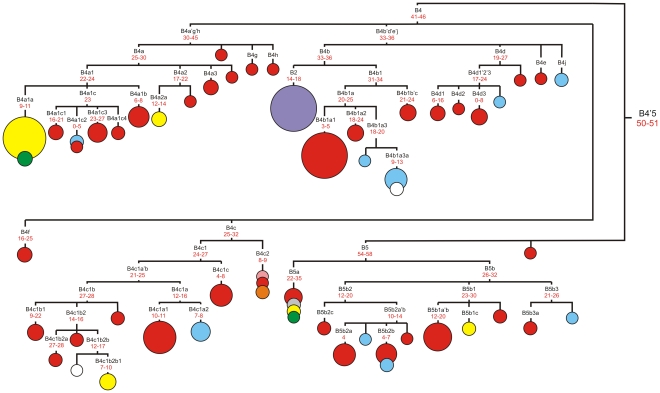
Complete mtDNA phylogenetic tree of haplogroup B4'B5. This schematic tree is based on phylogenetic tree presented in Figure S1. Time estimates (in kya) shown for mtDNA subclusters are based on the coding region substitutions [11], coding region synonymous substitutions [19] and complete genome substitutions [19]. The size of each circle is proportional to the number of individuals sharing the corresponding haplotype, with the smallest size corresponding to one individual. Geographical origin is indicated by different colors: northern Asian – in blue, central Asian – in pink, eastern Asian – in red, Indian – in grey, European – in white, Mainland southeastern Asian - in orange, Island southeastern Asian – in yellow, Oceania – in green, and Native American – in purple.

Noteworthy, the addition of a substantial set of completely sequenced mtDNAs from northern Asian populations has allowed us to reveal several new subclusters within the haplogroup B4 showing predominantly northern Asian distribution, *i.e.* B4b1a3, B4c1a2 and B4j (Figure 3, Figure S1). For example, identical Khamnigan and Buryat samples (Khm_21 and Br_336) bearing variants 16223 and 16362 as well as a series of specific mutations apparently belong to a previously unreported branch of haplogroup B–B4j, which is at the same phylogenetic level as nine other subclades (B4a–B4i) defined previously within B4 [18]. Ten of the new and one previously published sequence (Tubalar from southern Siberia [27]) clustered into uncommon B4b1a-branch, named B4b1a3, harboring the control region diagnostic motif 146-16086 (Figure S1). With the exception of Tubalar mtDNA having additional coding region transition at np 15007, all other B4b1a3 mtDNAs are characterized by 408A-9055-9388T-9615 motif defining subcluster B4b1a3a, which in turn can be further subdivided into two sister subclusters. The relatively large amount of internal variation accumulated in the northern Asian branch of B4b1a would mean that B4b1a3 arose *in situ* in southern Siberia after the arrival of B4b1a3 founder mtDNA from somewhere else in eastern Asia. The phylogeny depicted in Figure S1 provides additional information concerning the entry time of the founder mtDNA - the age of B4b1a3 node is estimated as ∼18–20 kya using different mutation rates, thus pointing to a pre-LGM/LGM, and apparently before the Holocene origin of this subcluster (Table S3).

Inside haplogroup B4 one more novel subgroup, B4c1a2, specific for northern Asian populations has been revealed (Figure 3, Figure S1). It is characterized by transition at np 16527 and back mutation at np 16311 which is together with transition at np 3497 thought to be diagnostic for a whole subclade B4c1 [18]. Subgroup B4c1a2 dates to 6–8 kya, demonstrating the Holocene time of divergence, like neighbouring eastern Asian specific subcluster B4c1a1, which is characterized by slightly older coalescence time estimated as 9.5–11 kya (Figure 3, Table S3). The remaining completely sequenced haplogroup B mtDNA lineages identified in the present work belong to different branches of B4 and B5 subgroups. Thus, Barghut sample (Bt_67) bears B4d1 diagnostic mutation at np 15038, whereas Buryat (Br_301) and Khamnigan (Khm_1) mtDNAs share variants 207 and 15758, suggesting their status as haplogroup B5b2b, which is distributed exclusively in eastern Asia; likewise, Altaian sample (Alt_196) is assigned into eastern Asian subgroup B5b*. It is intriguing that unique haplogroup B mtDNA variant revealed in eastern European Chuvashes (CT_45) precedes subcluster B4c1b2b1, which is characteristic for some Island southeastern Asian populations (Figure S1). Meanwhile, the remaining B-haplotypes detected in Chuvashes belong to southern Siberian subcluster B4b1a3a1a, pointing to Siberian ancestry for some maternal lineages in eastern European ethnic groups.

It should be noted that we have not found in northern Asia any haplogroup B mtDNA lineages ancestral to Amerindian-specific B2 branch. The only Tubalar mtDNA described previously by Starikovskaya et al. [27], designated there as B1 and interpreted as “closely related to Amerindian-specific B2 branch”, belongs in fact to the northern Asian-specific subcluster B4b1a3 (Figure S1) which in turns is a part of major subcluster B4b1, distributed predominantly in eastern Asia. Thus, there is no evidence at this time for the occurrence of haplogroup B2 mtDNA ancestors in Siberia, in contrast to the situation for haplogroup A2 and D2 mtDNAs [4], [8], [12], [29].

#### Haplogroup R9c

Haplogroup R9c1 is rare in eastern Asia (<0.5% in China), Mainland southeastern Asia (<1%), Taiwan (1.8%) and Island southeastern Asia (<3%), but appears at greater frequencies in the Philippines (3.3–5.7%) and Abor (11.1%) [23], [24], [30]. Notably, all R9c1 HVS1 variants described so far have a characteristic mutation at np 16157. The complete mtDNA sequence analysis shows that the lineages with this mutation belong to R9c1a1 subclade of haplogroup R9c1a (Figure S2). The most ancestral sequence (Bt_120) belonging probably to other R9c1a subclade indicates that R9c1a lineages could have been in the eastern Asia since 30–37 kya, and that the lineages, belonging to the R9c1a1 subgroup, participated in a more recent southeastern Asian expansion around 9 kya (Table S3), similar to that estimated for B4c2 [24] and E1a2 haplogroups [31].

Other haplogroup shared by eastern Asians and Mainland southeastern Asians is F2. This haplogroup has a slightly higher frequency in China (1.9–3.3%) and Thailand (2.4–5.4%) [30], [32] compared to the Laos (0.5%) [33], Taiwan (0.5%) [30], Vietnam (0.7%) and Formosa (0.1%) [24]. It should be noted that the majority of F2 HVS1 haplotypes revealed so far in eastern and southeastern Asia exhibit a base change at np 16291 whereas the single F2 sequence found in Barghuts bears a characteristic mutation at np 16260. The complete mtDNA sequence analysis shows that this variant (sample Bt_124) apparently belongs to a previously unreported branch of haplogroup F2 which we propose to label as F2e (Figure S2).

#### Haplogroup N9a

Haplogroup N9a is characteristic of eastern Asian populations, where it is detected at a highest frequencies in Japan (4.6%), China (2.8%), Mongolia (2.1%) and Korea (3.9%) [8], [21], [32], [34]. Haplogroup N9a is rare in Taiwan (1.2%) and Island southeastern Asia (1.1%) [22], [30], but appears at greater frequencies in Mainland southeastern Asia (1.5–4.5%) [24], [33]. With the comparable frequencies this haplogroup is detected in several populations of northern (0.9%–4.6%) and central Asia (1.2–2.5%), but it is virtually absent in western and southern Asia [8], [32], [35], [36]. Interestingly, haplogroup N9a is rarely found in the Volga-Ural region Tatars (∼1%) and Bashkirs (1.5%) as well as in some eastern Europeans, like Russians from southwestern Russia (1.5%) and Czechs (0.6%) [37]–[40].

In the current study we have reconstructed the phylogeny of haplogroup N9a based on 59 complete mtDNA genomes including ten newly sequenced samples and revised the classification of this haplogroup that was defined earlier as having seven main branches – N9a1'3; N9a2'4'5; N9a6–N9a10 [18]. Information from complete mtDNA sequencing reveals that Buryat sample (Br_623) and previously published Japanese sample (HNsq0240) from Tanaka et al. [21] share mutations at nps 11368 and 15090 and therefore belong to a rare N9a8 haplogroup (Figure S3). It should be noted that these two sequences showed deep divergence with each other being characterized by unique sets of seven and six mutations respectively. As follows from phylogenetic analysis data, our Barghut sample (Bt_81) shares transversions at nps 4668 and 5553 with two published Japanese samples [21] and therefore can be ascribed to a previously reported subcluster N9a2a3, Tatar sample (Tat_411G) which is identical to Japanese sample KAsq0018 [21] is a part of N9a2a2, Khamnigan (Khm_36) and Korean (Kor_87) mtDNAs belong to N9a1, whereas Korean (Kor_92) and Buryat (Br_433) variants can be identified as members of N9a3. Interestingly, Russian (Rus_BGII-19) and Czech (CZ_V-44) samples bearing transitions at nps 4913 and 12636 apparently belongs to a new subbranch N9a3a within haplogroup N9a3. Despite the low coalescence time estimates obtained for N9a3a (∼1.3–2.3 kya) it is quite probable that its founder had been introduced into eastern Europe much earlier taking into account the age of a whole N9a3 estimated as 8–13 kya and the discovery of a N9a haplotypes in a Neolithic skeletons from several sites, located in Hungary and belonged to the Körös Culture and Alföld Linear Pottery Culture, which appeared in eastern Hungary in the early 8th millennium B.P. [41], [42].

#### Haplogroups M10, M11 and M13

Haplogroups M10, M11 and M13 are most common in eastern Asia where they all detected at low frequencies (<5%) [8], [28], [32], [43]–[46]. Sporadically these haplogroups have been reported in southern, northern, central and southeastern Asia [8], [21], [30], [32], [36], [47], [48] as well as in eastern Europe – in Russians [49] and Kalmyks [8]. To further elucidate the origin of eastern Eurasian lineages found in mitochondrial gene pools of northern Asians and define more exactly the phylogeny of these rare haplogroups, we have completely sequenced mitochondrial genomes of ten individuals from populations of northern and eastern Asia, and eastern Europe (Figures S4, S5, and S6).

Until now there were only ten completely sequenced M10 subjects. The addition of our Shor sequence (Sh_27) to the tree (Figure S4) gives a branching point for M10a1, defined now by the only transition at np 16129. An Altaian sample (Alt_164) nested with Japanese sample (SCsq0008 [50]) formed a subclade, M10a1a2a, characterized by coding region mutation at np 10529 and back mutation at np 16129. Interestingly, our eastern European M10 mtDNAs (Rus_Vo-78 and Km_27) together with Japanese sequence (ONsq0096 [21]) clustered into another branch, M10a2a, within the second major M10a-subclade, M10a2. It should be noted that the results of mtDNA control region study in central Asian populations demonstrate the presence of M10a2a-haplotypes in Kazakhs at frequency of 0.8% [36]. In general, coalescence time estimate for M10a2a corresponds to 6–11 kya (Table S3), suggesting a relatively recent (post-Neolithic or later) origin and diffusion of M10a2a lineages from central Asia to eastern Europe.

We have also sequenced three complete M11 Siberian mtDNA genomes and compared them with all published M11 complete sequences. Figure S5 displays the reconstructed phylogeny of this haplogroup from which follows that our Buryat sequence (Br_444) fell into subhaplogroup M11a, whereas Altaian mtDNA genome (Alt_33) shared insertion of cytosine at np 459 and transition at np 5192 with Japanese mtDNA (HO1019 [51]) and formed a separate subclade, M11b2, within subhaplogroup M11b. It should be noted that one more subclade, M11b1, characterized by one control region (146) and two coding region (10685 and 14790) transitions can be revealed within M11b. Interestingly, a single M11 mtDNA sequence found in our Teleut samples (Tel_20) looks highly divergent being characterized by unique set of twelve mutations and belongs probably to a previously unreported branch of haplogroup M11, which we propose to designate as M11d.

As has been reported earlier haplogroup M13 encompass/encompasses two major subclades: M13a and M13b [18]. While subhaplogroup M13a was widely presented in eastern Asia and reached its greatest frequency and diversity in Tibet [45], [46], lineage M13b is restrictedly distributed in aboriginal populations of Malay Peninsula [47] and India [48]. In addition, subhaplogroup M13a has been detected at very low frequencies (∼1%) in southern Siberian Buryats and Khamnigans [8] and central Asian Kirghizs [36] as well as in Barghuts studied here. Phylogenetic analysis showed that our Buryat (Br_389) and Barghut (Bt_43) samples shared transition at np 5045 and formed a separate branch within eastern Asian-specific subhaplogroup M13a1b (Figure S6). A coalescence time estimate for subcluster M13a1b corresponds to 3–5 kya, suggesting a relatively recent (late Holocene or later) expansion of this lineage in eastern Asia and even more recent arrival of the M13a1b mtDNAs into northern Asia.

#### Haplogroup M9

Eastern Eurasian haplogroup M9 encompasses two subclades - E and M9a'b, showing a very distinctive geographic distribution. While subhaplogroup E is detected mainly in Island southeastern Asia and Taiwan, haplogroup M9a'b is distributed widely in mainland eastern Asia and Japan and relatively concentrated in Tibet and surrounding regions, including Nepal and northeastern India [31], [45], [46], [48], [52], [53]. It has been proposed recently that haplogroup M9 as a whole had most likely originated in southeastern Asia approximately 50 kya, whereas M9a'b itself spread northward into the eastern Asian mainland about 15 kya, after the LGM [31]. The complete mtDNA sequence analysis and the coalescence time estimates obtained suggest that certain subclades of M9a'b were likely associated with some post-LGM dispersals in eastern Asia, especially in Tibet [31], [45], [46], [53].

To further assess the variability of haplogroup M9a'b mtDNAs found in mitochondrial gene pools of eastern and northern Asians we have completely sequenced ten M9a samples representing Mongolians, Koreans, Kalmyks, Altaian Kazakhs, Khamnigans and Tuvinians (Table S4). Combining all published haplogroup M9a'b mtDNA genomes and our newly collected samples, we reconstructed a tree of 132 complete sequences (Figure S7). According to this updated phylogenetic tree, we have not found any northern Asian-specific subclades of M9a, but we were able to efficiently allocate our new M9a variants into already defined and some newly identified subclades of this haplogroup (Figure S7). For instance, our Korean (Kor_30), Mongolian (Mn_16) and Kalmyk (Km_68) samples appear as singletons within major subclades M9a1, M9a1b1 and M9a1a1a1, respectively. Meanwhile, Altaian Kazakh (Kz_69) and Kalmyk (Km_79) samples bear transversion at np 10951 and belong to subcluster M9a1b2 revealed recently in southwestern Chinese representatives [53], whereas Korean (Kor_10) mtDNA and complete genome of Vietnamese individual (Kinh_88 [53]) share transition at np 6815 and may therefore represent a new subcluster, M9a4b, within M9a4, distributed both in southeastern Asia and southern and northern China (Figure S7). Interestingly, the remaining of our M9a mtDNA sequences (Br_377, Khm_15, Tv_351c) fall into subclades which were mainly found in Japan (M9a1a1a1), Japan and China (M9a1a1c1a1), southwestern China and Tibet (M9a1a1c1b). Thus, the M9a1a1-lineages revealed in northern Asian populations could be regarded as a traces of northward Late Glacial dispersal(s) originating in southern China about 14–17 kya proposed on the basis of the phylogeographic pattern of haplogroup M9a1a1 [53].

### Conclusions

In order to achieve a thorough coverage of DNA lineages revealed in the northern Asian matrilineal gene pool, we have completely sequenced the mtDNA of 55 samples representing haplogroups R11, B4, B5, F2, M9, M10, M11, M13, N9a and R9c1, which were pinpointed from a massive collection of northern and eastern Asian, as well as European control region mtDNA sequences. By comparing with the all available complete mtDNA sequences, these mtDNAs have been assigned into the available haplogroups with a number of novel lineages identified from a comprehensive phylogenetic analysis.

Overall, the new data confirm that the dissection of mtDNA haplogroups into subhaplogroups of younger age and more limited geographic and ethnic distributions might reveal previously unidentified spatial frequency patterns, which could be further correlated to prehistoric and historical migratory events. Thus, the addition of a large number of completely sequenced haplogroup B mtDNAs from northern and eastern Asian populations to available data sets has allowed us to reveal a few new subclusters within the haplogroup B4 (B4b1a3, B4b1a3a, B4c1a2 and B4j) showing predominantly northern Asian distribution. The whole subcluster B4b1a3 showed a coalescent time of approximately 18 to 20 kya, whereas subclusters B4b1a3a and B4c1a3 emerged around 9 to 13 kya and 7 to 8 kya, respectively. As a result, coalescence age estimates placed the origin of subcluster B4b1a3 in the LGM episode, while subclusters B4b1a3a and B4c1a2 are in a more recent post-glacial period (the end of the Pleistocene and the early Holocene). Our findings confirm our previous conclusion that northern Asian maternal gene pool consists of predominantly post-LGM components of eastern Asian ancestry, though some genetic lineages may have a pre-LGM/LGM origin [12].

Notably, the observation that the most ancestral B4b1a3-sequence preceding subcluster B4b1a3a, as well as some of our newly recognized highly divergent mtDNA haplotypes (*i.e.* within subclusters R11b, M10a1 and M11d) originated from Altai region of southern Siberia, further suggested that the southern mountain belt of Siberia acts as a likely main route for pioneer settlement of northern Asia [54]–[57].

The results of our study provided an additional support for the existence of limited maternal gene flow between eastern Asia/southern Siberia and eastern Europe revealed by analysis of modern and ancient mtDNAs previously [12], [37], [39], [48], [42], [58], [59]. Two more mtDNA subclusters which may be indicative of eastern Asian influx into gene pool of eastern Europeans have been revealed within haplogroups M10 and N9a. The presence of N9a3a subcluster only in eastern European populations may indicate that it could arose there after the arrival of founder mtDNA from eastern Asia about 8–13 kya. It is noteworthy that another eastern Asian specific lineage, C5c1, revealed exclusively in some European populations (Poles, Belorussians, Romanians), shows evolutionary ages within frames of 6.6–11.8 kya depending on the mutation rates values [12]. In addition, recent molecular-genetic study of the Neolithic skeletons from archaeological sites in the Alföld (Hungary) has demonstrated high frequency of eastern Asian mtDNA haplogroups in ancient inhabitants of the Carpathian Basin [42]. Specifically, haplogroups N9a and C5 were also revealed in remains, thus indicating that genetic continuity for some eastern Asian mtDNA lineages in Europeans is possible from the Neolithic Period. Prehistoric migrations associated with the distribution of the pottery-making tradition initially emerged in the forest-steppe belt of northern Eurasia starting at about 16 kya and spread to the west to reach the south-eastern confines of eastern European Plain by about 8 kya [60] could be suggested as a potential cause for eastern Asian mtDNA haplogroups appearance in Europe. More information from complete mtDNA sequences as well as the other genetic markers in the contemporary and extinct populations of Eurasia would be helpful to validate our conclusions.

## Materials and Methods

### Ethics Statement

The study was approved by Bioethics Committee of the Nicolaus Copernicus University in Torun, The Ludwik Rydygier Collegium in Bydoszcz, Poland (statements no. KB/32/2002 and KB/414/2008 from 28 January, 2002 and 17 September, 2008, respectively). All subjects provided written informed consent for the collection of samples and subsequent analysis.

### Sampling, HVS1 Sequencing and RFLP Typing

Blood samples from 149 unrelated Barghuts were collected in different localities of Hulun Buir Aimak, Inner Mongolia, China. Hair samples from 98 unrelated Altaian Kazakhs were collected in different localities of Kosh-Agach district of Altai Republic. Total DNA was extracted by the standard phenol/chloroform method. The hypervariable segments (HVS1) (from positions 15999 to 16400) and HVS2 (from positions 30 to 407) were sequenced in all samples followed by RFLP screening to resolve haplogroup status in a hierarchical scheme as described earlier [8].

### Complete mtDNA Sequencing

For complete mtDNA sequencing we have choose the mtDNA lineages which are specific for populations of northern Asia but which are still underrepresented in the published data sets on complete mtDNA variation (haplogroup B) as well as other eastern Eurasian mtDNA haplogroups which are rarely found in populations of northern Asia (R11, F2, M9, M10, M11, M13, N9a and R9c1) being much more frequent in other regions of Asia. Out of about 5000 samples of northern and eastern Asians (including 247 samples presented here) as well as Europeans that had been screened previously for haplogroup-diagnostic RFLP markers and subjected to control region sequencing [8], [38]–[40], [49], [61]–[67] (Table S5) a total of 55 samples representing haplogroups B (n = 23), F2 (n = 1), M9 (n = 9), M10 (n = 5), M11 (n = 3), M13 (n = 2), N9a (n = 10), R9c (n = 1) and R11 (n = 1) were selected (Table S4). Complete mtDNA sequencing was performed using the methodology described in detail by Torroni et al. [68]. DNA sequence data were analyzed using SeqScape v. 2.5 software (Applied Biosystems) and compared with the revised Cambridge reference sequence (rCRS) [69].

### Data Analysis

Descriptive statistical indexes, the Tajima's *D* [70] and Fu's *F*S [71] neutrality tests (for HVS1 sequence data) were calculated using Arlequin software, version 3.01 [72]. Principal Component (PC) analysis was performed using mtDNA haplogroup frequencies as input vectors by STATISTICA 6.0 software (StatSoft, Inc., USA). Nonparametric multidimensional scaling (MDS) analysis based on F_ST_ statistics calculated from HVS1 sequences was also performed using STATISTICA 6.0 software (StatSoft, Inc., USA) to visualize relationships between Altaian Kazakhs and Barghuts studied and other Asian populations around. Published data on mtDNA diversity in western, eastern, central and northern Asian populations [8], [73]–[77] as well as in Mongolic-speaking Kalmyks [8] residing now in eastern Europe but descended from western Mongolians (Oirats) were included in our comparative analysis.

For reconstruction of the complete mtDNA phylogenies of haplogroups B, F2, M9, M10, M11, M13, N9a, R9c and R11 the data obtained in this study and those published previously [4], [5], [20]–[28], [44]–[48], [50]–[53], [58], [78]–[90] as well as FamilyTreeDNA project data available at PhyloTree [18], were taken into account. A nomenclature, which we hereby update, follows van Oven and Kayser [18], with several new modifications. The most-parsimonious trees of the complete mtDNA sequences were reconstructed manually, and verified by means of the Network 4.5.1.0 software [91], and using mtPhyl 2.8.0.0 software (http://eltsov.org), which is designed to reconstruct maximum parsimony phylogenetic trees. Both applications calculate haplogroup divergence estimates (ρ) and their error ranges, as average number of substitutions in mtDNA clusters (haplogroups) from the ancestral sequence type [92]. Values of mutation rates based on mtDNA complete genome variability data (one mutation every 3624 years [19]), coding region substitutions (one mutation every 4610 years [11]) and synonymous substitutions (one mutation every 7884 years [19]) were used.

Overall, 508 mitochondrial genomes – 242 B, 10 F2, 132 M9, 15 M10, 16 M11, 25 M13, 59 N9a, 4 R9c1 and 5 R11 – were analyzed. Nucleotide position (np) 16519 as well as positions showing point indels and/or transversions located between nps 16180–16193, 303–315, 522–524, 960–963 were excluded from the phylogenetic analysis. The GenBank accession numbers for the complete mitochondrial genomes reported in this paper are JN857009–JN857063.

## Supporting Information

Figure S1 **Phylogenetic tree of haplogroups R11'B6 and B4'B5 constructed using the program mtPhyl.** Numbers along links refer to substitutions scored relative to rCRS [69]. Transversions are further specified; ins and del denote insertions and deletions of nucleotides, respectively; back mutations are underlined; symbol<denotes parallel mutation. Sequences indicated in red print are new (Table S4) while the others have been taken from Ingman et al. [78]; Kong et al. [28]; Tanaka et al. [21]; Starikovskaya et al. [27]; Ueno et al. [86]; Tabbada et al. [23]; Kong et al. [89]; Bilal et al. [50]; Loo et al. [25]; Kazuno et al. [80]; Pierson et al. [26]; Hartmann et al. [85]; Thangaraj et al. [81]; Nohira et al. [51]; Kong et al. [44]; Tamm et al. [4], Achilli et al. [5], Just et al. [83]; Zou et al. [87]; Trejaut et al. [22]; Mishmar et al. [79]; Macaulay et al. [47]; Razafindrazaka et al. [82]; Peng et al. [24]; as well as FamilyTreeDNA project data available at PhyloTree [18]. The particular sequences from these sources are referred to as MI, QK, MT, ES, HU, KT, QPK, EB, JL, AK, MJP, AH, KTH, CN, QP, ET, AA, RJ, YZ, AT, DM, VM, HR, MSP and FTDNA respectively, followed by number sign (#) and the original sample code. Established haplogroup labels are shown in black; blue are redefined and red are newly identified haplogroups in the present study.
(XLSX)

Figure S2 **Phylogenetic tree of haplogroup R9c, constructed using the program mtPhyl.** Numbers along links refer to substitutions scored relative to rCRS [69]. Transversions are further specified; ins and del denote insertions and deletions of nucleotides, respectively; back mutations are underlined; symbol<denotes parallel mutation. Sequences indicated in red print are new (Table S4) while the others have been taken from Kong et al. [28]; Tanaka et al. [21]; Tabbada et al. [23]; Bilal et al. [50]; Gunnarsdottir et al. [88]; Wang et al. [90]; as well as FamilyTreeDNA project data available at PhyloTree [18]. The particular sequences from these sources are referred to as QK, MT, KT, EB, EG, CW, and FTDNA respectively, followed by number sign (#) and the original sample code. Established haplogroup labels are shown in black; blue are redefined and red are newly identified haplogroups in the present study.
(XLS)

Figure S3 **Phylogenetic tree of haplogroup N9a, constructed using the program mtPhyl.** Numbers along links refer to substitutions scored relative to rCRS [69]. Transversions are further specified; ins denotes insertions of nucleotides; back mutations are underlined; symbol<denotes parallel mutation. Sequences indicated in red print are new (Table S4) while the others have been taken from Kong et al. [28]; Tanaka et al. [21]; Ueno et al. [86]; Kazuno et al. [80]; as well as FamilyTreeDNA project data available at PhyloTree [18]. The particular sequences from these sources are referred to as QK, MT, HU, AK, and FTDNA respectively, followed by number sign (#) and the original sample code. Established haplogroup labels are shown in black; blue are redefined and red are newly identified haplogroups in the present study.
(XLS)

Figure S4 **Phylogenetic tree of haplogroup M10, constructed using the program mtPhyl.** Numbers along links refer to substitutions scored relative to rCRS [69]. Ins and del denote insertions and deletions of nucleotides, respectively; back mutations are underlined; symbol<denotes parallel mutation. Sequences indicated in red print are new (Table S4) while the others have been taken from Kong et al. [28]; Tanaka et al. [21]; Bilal et al. [50]; Kong et al. [44]; Chandrasekar et al. [48]. The particular sequences from these sources are referred to as QK, MT, EB, QP, and AC respectively, followed by number sign (#) and the original sample code. Established haplogroup labels are shown in black; blue are redefined and red are newly identified haplogroups in the present study.
(XLS)

Figure S5 **Phylogenetic tree of haplogroup M11, constructed using the program mtPhyl.** Numbers along links refer to substitutions scored relative to rCRS [69]. Transversions are further specified; ins denotes insertion of nucleotide; back mutations are underlined; symbol<denotes parallel mutation. Sequences indicated in red print are new (Table S4) while the others have been taken from Kong et al. [28]; Tanaka et al. [21]; Bilal et al. [50]; Nohira et al. [51]; Chandrasekar et al. [48], Qin et al. [46]; as well as FamilyTreeDNA project data available at PhyloTree [18]. The particular sequences from these sources are referred to as QK, MT, EB, CN, AC, ZQ and FTDNA respectively, followed by number sign (#) and the original sample code. Established haplogroup labels are shown in black; blue are redefined and red are newly identified haplogroups in the present study.
(XLS)

Figure S6 **Phylogenetic tree of haplogroup M13'46'61, constructed using the program mtPhyl.** Numbers along links refer to substitutions scored relative to rCRS [69]. Transversions are further specified; ins and del denote insertions and deletions of nucleotides, respectively; back mutations are underlined; symbol<denotes parallel mutation. Sequences indicated in red print are new (Table S4) while the others have been taken from Tanaka et al. [21]; Kong et al. [44]; Macaulay et al. [47], Dancause et al. [84]; Fornarino et al. [52]; Chandrasekar et al. [48], Qin et al. [46]; Zhao et al. [45]. The particular sequences from these sources are referred to as MT, QP, VM, KD, SF, AC, ZQ and MZ respectively, followed by number sign (#) and the original sample code. Established haplogroup labels are shown in black; blue are redefined and red are newly identified haplogroups in the present study.
(XLS)

Figure S7 **Phylogenetic tree of haplogroup M9a'b, constructed using the program mtPhyl.** Numbers along links refer to substitutions scored relative to rCRS [69]. Transversions are further specified; ins and del denote insertions and deletions of nucleotides, respectively; back mutations are underlined; symbol<denotes parallel mutation. Sequences indicated in red print are new (Table S4) while the others have been taken from Ingman et al. [78]; Kong et al. [28]; Tanaka et al. [21]; Ingman, Gyllensten [58]; Ueno et al. [86]; Chandrasekar et al. [48]; Bilal et al. [50]; Kong et al. [44]; Qin et al. [46]; Zhao et al. [45]; Peng et al. [53]; Soares et al. [20]. The particular sequences from these sources are referred to as MI, QK, MT, IG, HU, AC, EB, ZQ, MZ, MP, PS, respectively, followed by number sign (#) and the original sample code. Established haplogroup labels are shown in black; blue are redefined and red are newly identified haplogroups in the present study.
(XLS)

Table S1 **Control region sequences of 149 Barghut and 98 Altaian Kazakh mtDNA samples analyzed in the present study.** Samples which were selected for complete mtDNA sequencing are indicated in ““Compl. seq. ID”” column.
(XLS)

Table S2 **Population distribution and frequencies of haplogroup B and its subhaplogroups B2, B4 and B5.**
(XLS)

Table S3 **Estimated ages of selected subclasters of mtDNA haplogroups R11b, B4'B5, R9c, M9, M10, M11 and M13.**
(DOC)

Table S4 **Control-region variation of the completely sequenced mtDNAs belonging to haplogroups R11'B6, B4'B5, R9c, M9, M10, M11, M13 and N9a.**
(DOC)

Table S5 **List of population samples subjected previously for haplogroup-diagnostic RFLP screening and control region sequencing from where 55 samples were selected for complete mtDNA sequencing.**
(XLS)
