# Supplementary material for: Complete Mitochondrial DNA Analysis of Eastern Eurasian Haplogroups Rarely Found in Populations of Northern Asia and Eastern Europe
Source: PLoS One. 2012 Feb 21;7(2):e32179. doi: 10.1371/journal.pone.0032179 (PMC3283723; doi:10.1371/journal.pone.0032179)
Supplement: Table S3 — Estimated ages of selected subclasters of mtDNA haplogroups R11b, B4'B5, R9c, M9, M10, M11 and M13. (DOC) [file pone.0032179.s010.doc]

Table S3. Estimated ages of selected subclasters of mtDNA haplogroups R11b, B4’B5, R9c, M9, M10, M11 and M13.

| Haplogroup | n | Coding region substitutions [11] | | Coding region synonymous substitutions [19] | | Complete genome substitutions [19] | |
| --- | --- | --- | --- | --- | --- | --- | --- |
|  |  | ρ ± δ | T (ky) | ρ ± δ | T (ky) | ρ ± δ | T (ky) |
| B4b1a | 38 | 4.84 ± 1.33 | 22.32 ± 6.13 | 2.5 ± 0.94 | 19.71 ± 7.44 | 9 ± 1.82 | 24.81 (14.56, 35.52) |
| **B4b1a3** | 11 | 3.91 ± 1.58 | 18.02 ± 7.27 | 2.55 ± 1.27 | 20.07 ± 10.03 | 7.09 ± 2.18 | 19.27 (7.41, 31.83) |
| **B4b1a3a** | 10 | 2.2 ± 1 | 10.14 ± 4.61 | 1.7 ± 0.97 | 13.4 ± 7.68 | 3.7 ± 1.32 | 9.79 (2.9, 16.96) |
| **B4b1a3a1** | 9 | 1 ± 0.43 | 4.61 ± 1.98 | 0.78 ± 0.4 | 6.13 ± 3.16 | 2.44 ± 1.03 | 6.4 (1.09, 11.89) |
| B4b’d’e’j | 79 | 9.27 ± 1.68 | 42.72 ± 7.77 | 6.16 ± 1.55 | 48.6 ± 12.18 | 14.42 ± 2.11 | 41.21 (28.6, 54.35) |
| B4d1 | 3 | 1.33 ± 0.67 | 6.15 ± 3.07 | 2 ± 0.82 | 15.77 ± 6.44 | 5 ± 1.37 | 13.37 (6.03, 21.01) |
| B4c1b2 | 7 | 3 ± 0.85 | 13.83 ± 3.9 | 1.86 ± 0.74 | 14.64 ± 5.85 | 6 ± 1.4 | 16.17 (8.59, 24.06) |
| B4c1b2b | 5 | 2.8 ± 1.06 | 12.91 ± 4.88 | 2.2 ± 1 | 17.34 ± 7.88 | 4.6 ± 1.22 | 12.26 (5.79, 18.97) |
| B4c1a | 22 | 2.68 ± 0.86 | 12.36 ± 3.96 | 2 ± 0.81 | 15.77 ± 6.41 | 4.91 ± 1.09 | 13.12 (7.26, 19.16) |
| **B4c1a2** | 6 | 1.67 ± 0.58 | 7.68 ± 2.66 | 0.83 ± 0.37 | 6.57 ± 2.94 | 2.67 ± 0.75 | 7 (3.12, 10.96) |
| B5b2b | 9 | 1.56 ± 0.63 | 7.17 ± 2.9 | 0.56 ± 0.25 | 4.38 ± 1.96 | 2.56 ± 0.81 | 6.7 (2.51, 11) |
| B5b3 | 3 | 5.33 ± 1.63 | 24.59 ± 7.53 | 3.33 ± 1.33 | 26.28 ± 10.51 | 7.67 ± 1.91 | 20.93 (10.37, 32.01) |
| R11 | 5 | 5 ± 1.25 | 23.05 ± 5.76 | 2.4 ± 0.94 | 18.92 ± 7.4 | 7.8 ± 1.48 | 21.31 (13.07, 29.87) |
| R11b | 3 | 3.67 ± 1.11 | 16.9 ± 5.1 | 1.67 ± 0.75 | 13.14 ± 5.88 | 7.33 ± 1.63 | 19.97 (10.97, 29.35) |
| R9c1a | 4 | 7.25 ± 2.02 | 33.42 ± 9.29 | 4.75 ± 1.64 | 37.45 ± 12.92 | 11 ± 2.4 | 30.75 (17.02, 45.23) |
| R9c1a1 | 3 | 2 ± 0.82 | 9.22 ± 3.76 | 1 ± 0.58 | 7.88 ± 4.55 | 3.33 ± 1.05 | 8.79 (3.28, 14.48) |
| M11 | 16 | 5.06 ± 1.22 | 23.34 ± 5.62 | 2.81 ± 0.99 | 22.17 ± 7.78 | 8.31 ± 1.48 | 22.8 (14.55, 31.36) |
| M11a’b | 14 | 4.21 ± 0.95 | 19.43 ± 4.38 | 1.93 ± 0.5 | 15.2 ± 3.94 | 7.14 ± 1.32 | 19.42 (12.13, 26.97) |
| M11a | 10 | 3.3 ± 0.66 | 15.21 ± 3.02 | 1.6 ± 0.47 | 12.61 ± 3.7 | 5.7 ± 0.99 | 15.33 (9.93, 20.88) |
| M11a1 | 3 | 2 ± 0.94 | 9.22 ± 4.35 | 0.67 ± 0.47 | 5.26 ± 3.72 | 2.67 ± 1.05 | 7 (1.55, 12.63) |
| M11a2 | 4 | 4.25 ± 1.09 | 19.59 ± 5.02 | 1.5 ± 0.61 | 11.83 ± 4.83 | 6.5 ± 1.5 | 17.59 (9.41, 26.1) |
| M11b | 4 | 3 ± 1.06 | 13.83 ± 4.89 | 1.75 ± 0.83 | 13.8 ± 6.54 | 4.75 ± 1.3 | 12.68 (5.76, 19.86) |
| M10 | 15 | 7.93 ± 2.04 | 36.57 ± 9.4 | 4.73 ± 1.68 | 37.32 ± 13.22 | 12 ± 2.33 | 33.77 (20.26, 47.97) |
| M10a | 14 | 4.14 ± 0.86 | 19.1 ± 3.98 | 1.86 ± 0.46 | 14.64 ± 3.65 | 8.36 ± 1.48 | 22.93 (14.65, 31.52) |
| M10a1 | 8 | 5.25 ± 1.39 | 24.2 ± 6.42 | 2.25 ± 0.73 | 17.74 ± 5.75 | 10.25 ± 2.19 | 28.51 (16.07, 41.58) |
| M01a1a | 7 | 4.29 ± 1.2 | 19.76 ± 5.51 | 2.43 ± 0.82 | 19.15 ± 6.47 | 6.57 ± 1.44 | 17.79 (9.91, 25.98) |
| M10a1a1 | 4 | 1.75 ± 0.75 | 8.07 ± 3.46 | 1.25 ± 0.66 | 9.86 ± 5.21 | 2.75 ± 0.97 | 7.22 (2.2, 12.39) |
| M10a1a2 | 3 | 3.67 ± 1.2 | 16.9 ± 5.54 | 2.67 ± 1.05 | 21.02 ± 8.31 | 5.67 ± 1.53 | 15.23 (7.01, 23.81) |
| M10a2 | 5 | 2.6 ± 0.87 | 11.99 ± 4.02 | 1.4 ± 0.53 | 11.04 ± 4.17 | 3.6 ± 1.1 | 9.52 (3.77, 15.46) |
| M10a2a | 3 | 1.67 ± 0.75 | 7.68 ± 3.44 | 1.33 ± 0.67 | 10.51 ± 5.26 | 2.33 ± 0.88 | 6.1 (1.56, 10.78) |
| M13 | 20 | 9.2 ± 1.9 | 42.41 ± 8.74 | 4.45 ± 1.22 | 35.08 ± 9.65 | 16.2 ± 2.48 | 46.81 (31.75, 62.56) |
| M13a | 14 | 4 ± 1.09 | 18.44 ± 5.04 | 1.71 ± 0.63 | 13.52 ± 4.97 | 7.71 ± 1.56 | 21.07 (12.43, 30.06) |
| M13a1 | 9 | 1.67 ± 0.71 | 7.68 ± 3.28 | 0.89 ± 0.52 | 7.01 ± 4.11 | 3.44 ± 1.06 | 9.09 (3.55, 14.83) |
| M13a1a | 4 | 0.75 ± 0.43 | 3.46 ± 2 | 0.25 ± 0.25 | 1.97 ± 1.97 | 1.5 ± 0.61 | 3.9 (0.77, 7.09) |
| M13a1b | 5 | 0.8 ± 0.49 | 3.69 ± 2.26 | 0.6 ± 0.45 | 4.73 ± 3.53 | 1.6 ± 0.69 | 4.16 (0.62, 7.78) |
| M13a2 | 5 | 2.4 ± 0.69 | 11.06 ± 3.19 | 1.2 ± 0.49 | 9.46 ± 3.86 | 3.8 ± 0.96 | 10.06 (5, 15.27) |
| M13b | 6 | 6.67 ± 1.8 | 30.73 ± 8.28 | 5.17 ± 1.61 | 40.73 ± 12.67 | 11.33 ± 2.39 | 31.75 (18, 46.25) |
| M13b2 | 4 | 2 ± 1.41 | 9.22 ± 6.52 | 2 ± 1.41 | 15.77 ± 11.15 | 3 ± 1.73 | 7.89 (-1.01, 17.29) |
| M9a1a | 77 | 4.3 ± 1.21 | 19.82 ± 5.59 | 2.56 ± 0.99 | 20.17 ± 7.78 | 6.31 ± 1.3 | 17.05 (9.96, 24.4) |
| M9a1a1a1 | 15 | 1 ± 0.26 | 4.61 ± 1.19 | 0.8 ± 0.23 | 6.31 ± 1.82 | 1.4 ± 0.35 | 3.63 (1.86, 5.43) |
| M9a1a1c1a1 | 9 | 1.11 ± 0.35 | 5.12 ± 1.62 | 0.67 ± 0.27 | 5.26 ± 2.15 | 2.11 ± 0.48 | 5.51 (3.01, 8.05) |
| M9a1a1c1b1 | 28 | 1.82 ± 0.78 | 8.4 ± 3.6 | 0.61 ± 0.16 | 4.79 ± 1.23 | 3.68 ± 1.21 | 9.73 (3.4, 16.3) |
| M9a1b1 | 29 | 2.31 ± 0.57 | 10.65 ± 2.65 | 1.34 ± 0.44 | 10.6 ± 3.45 | 3.62 ± 0.66 | 9.57 (6.11,13.11) |
| M9a1b2 | 4 | 0.5 ± 0.35 | 2.31 ± 1.63 | 0.25 ± 0.25 | 1.97 ± 1.97 | 1.5 ± 0.71 | 3.9 (0.29, 7.58) |
| M9a4 | 8 | 3.12 ± 0.8 | 14.41 ± 3.69 | 1.5 ± 0.47 | 11.83 ± 3.69 | 5 ± 1.17 | 13.37 (7.09, 19.87) |

Subclusters specific for northern Asian populations are shown in bold.
