# Supplementary material for: Complete Mitochondrial DNA Analysis of Eastern Eurasian Haplogroups Rarely Found in Populations of Northern Asia and Eastern Europe
Source: PLoS One. 2012 Feb 21;7(2):e32179. doi: 10.1371/journal.pone.0032179 (PMC3283723; doi:10.1371/journal.pone.0032179)
Supplement: Table S4 — Control-region variation of the completely sequenced mtDNAs belonging to haplogroups R11'B6, B4'B5, R9c, M9, M10, M11, M13 and N9a. (DOC) [file pone.0032179.s011.doc]

Table S4. Control-region variation of the 55 completely sequenced mtDNAs

| Sample | Haplogroup | HVS1  (minus 16000) | HVS2 | Population | Sample location |
| --- | --- | --- | --- | --- | --- |
| Br_417 | B4b1a3a | 136 183AC 189 217 | 73 263 309.1C 315.1C | Buryat | Russia, South Siberia, Buryat Republic |
| Alt_124 | B4b1a3a1 | 086 136 182AC 183AC 189 217 | 73 146 263 309.1C 309.2C 315.1C | Altaian-Kizhi | Russia, South Siberia, Altai Republic |
| Alt_175 | B4b1a3a1 | 086 136 182AC 183AC 189 217 | 73 146 207 263 309.1C 309.2C 315.1C | Altaian-Kizhi | Russia, South Siberia, Altai Republic |
| Br_604 | B4b1a3a1 | 086 136 183AC 189 217 | 73 146 207 263 309.1C 315.1C | Buryat | Russia, South Siberia, Buryat Republic |
| Khm_44 | B4b1a3a1 | 086 136 183AC 189 193.1C 217 327 | 73 146 207 263 309.1C 315.1C | Khamnigan | Russia, South Siberia, Buryat Republic |
| Khm_62 | B4b1a3a1 | 086 136 183AC 189 217 | 73 146 207 263 309.1C 309.2C 315.1C | Khamnigan | Russia, South Siberia, Buryat Republic |
| Khm_52 | B4b1a3a1 | 086 136 183AC 189 217 | 73 146 207 263 309.1C 315.1C | Khamnigan | Russia, South Siberia, Buryat Republic |
| Sh_15' | B4b1a3a1 | 086 136 183AC 189 217 | 73 146 207 263 309.1C 315.1C | Shor | Russia, South Siberia, Kemerovo region |
| CT_53 | B4b1a3a1 | 086 136 183AC 189 217 | 73 146 207 263 315.1C | Chuvash | Eastern Europe, Russia, Volga-Ural Region, Chuvash Republic |
| CT_55 | B4b1a3a1 | 086 136 183AC 189 217 | 73 146 207 263 315.1C | Chuvash | Eastern Europe, Russia, Volga-Ural Region, Chuvash Republic |
| Yak_43 | B4c1a2 | 176 182AC 183AC 189 217 | 73 263 315.1C | Yakut | Russia, Central Siberia, Yakut (Sakha) Republic |
| Br_570 | B4c1a2 | 126 C/T 182AC 183AC 189 217 | 73 263 309.1C 309.2C 315.1C | Buryat | Russia, South Siberia, Buryat Republic |
| Br_408 | B4c1a2 | 182AC 183AC 189 217 | 73 263 309.1C 309.2C 315.1C | Buryat | Russia, South Siberia, Buryat Republic |
| Br_383 | B4c1a2 | 182AC 183AC 189 217 | 73 263 309.1C 309.2C 309.3C 315.1C | Buryat | Russia, South Siberia, Buryat Republic |
| Khm_69 | B4c1a2a | 092 182AC 183AC 189 217 319 | 73 263 309.1C 309.2C 315.1C | Khamnigan | Russia, South Siberia, Buryat Republic |
| Bt_94 | B4c1a2a | 092 182AC 183AC 189 217 | 73 263 309.1C 309.2C 315.1C | Barghut | China, Inner Mongolia, Hulun Buir Aimak |
| CT_45 | B4c1b2b | 140 178 182AC 183AC 189  217 274 291 3355 | 73 146 150 189 195 263 309.1C 315.1C | Chuvash | Eastern Europe, Russia, Volga-Ural Region, Chuvash Republic |
| Bt_67 | B4d1 | 183AC 189 217 240 | 73 152 263 309.1C 309.2C 315.1C | Barghut | China, Inner Mongolia, Hulun Buir Aimak |
| Khm_21 | B4j | 183AC 189 217 223 362 | 73 263 309.1C 315.1C | Khamnigan | Russia, South Siberia, Buryat Republic |
| Br_336 | B4j | 183AC 189 217 223 362 | 73 263 309.1C 309.2C 315.1C | Buryat | Russia, South Siberia, Buryat Republic |
| Br_301 | B5b2b | 051 111 140 145 189 234 243 | 73 131 204 207 263 309.1C 315.1C | Buryat | Russia, South Siberia, Buryat Republic |
| Khm_1 | B5b2b | 111 140 183AC 189 234 243 | 73 131 204 207 263 309.1C 315.1C | Khamnigan | Russia, South Siberia, Buryat Republic |
| Alt_196 | B5b* | 140 182AC 183AC 189 243 274 | 73 103 146 203 204 263 309.1C 309.2C 315.1 | Altaian-Kizhi | Russia, South Siberia, Altai Republic |
| Bt_124 | F2e | 260 304 | 67 73 153 249D 263 309.1C 315.1C | Barghut | China, Inner Mongolia, Hulun Buir Aimak |
| Sh_27 | M10a1* | 129 186 223 311 362 | 73 146 263 309.1C 315.1C | Shor | Russia, South Siberia, Kemerovo region |
| Mn_9 | M10a1a1 | 129 223 311 | 73 263 309.1C 315.1C | Mongolian | Mongolia |
| Alt_164 | M10a1a2a | 093 223 311 357 381 | 73 146 189 199 263 309.1C 315.1C | Altaian-Kizhi | Russia, South Siberia, Altai Republic |
| Km_27 | M10a2a | 066 223 311 | 73 200 263 315.1C | Kalmyk | Russia, Kalmyk Republic |
| Rus_Vo-78 | M10a2a | 066 145 223 311 | 73 200 263 315.1C | Russian | Russia, Novgorod region |
| Br_444 | M11a | 223 | 73 198 200 215 263 309.1C 315.1C 318 326 | Buryat | Russia, South Siberia, Buryat Republic |
| Alt_33* | M11b2 | 223 | 73 200 215 263 309.1C 315.1C 318 326 | Altaian-Kizhi | Russia, South Siberia, Altai Republic |
| Tel_20 | M11d | 189 223 295 | 64 65 73 188 200 215 263 309.1C 315.1C 318 326 | Teleut | Russia, South Siberia, Novokuznetsk region |
| Bt_43 | M13a1b | 145 148 188 189 223 381 | 73 152 263 315.1C | Barghut | China, Inner Mongolia, Hulun Buir Aimak |
| Br_389 | M13a1b | 086 145 148 188 189 223 381 | 73 152 263 315.1C | Buryat | Russia, South Siberia, Buryat Republic |
| Kor_30 | M9a1a* | 223 234 248 265AC 316 362 | 73 153 263 309.1C 315.1C | Korean | South Korea, Seoul |
| Khm_15 | M9a1a1a1 | 223 234 316 362 | 73 195 263 309.1C 315.1C | Khamnigan | Russia, South Siberia, Buryat Republic |
| Km_68 | M9a1a1a1* | 223 234 316 362 | 73 263 309.1C 315.1C | Kalmyk | Russia, Kalmyk Republic |
| Tv_351c | M9a1a1c1a1 | 223 234 291 316 362 | 73 153 263 309.1C 309.2C 315.1C | Tuvinian | Russia, South Siberia, Tyva Republic |
| Br_377 | M9a1a1c1b | 166 189 223 234 316 362 | 73 263 315.1C | Buryat | Russia, South Siberia, Buryat Republic |
| Mn_16 | M9a1b1* | 158 223 224 234 362 | 73 150 152 263 309.1C 315.1C | Mongolian | Mongolia |
| Kz_69 | M9a1b2 | 223 362 | 73 150 152 153 195 263 309.1C 315.1C | Altaian Kazakh | Russia, South Siberia, Altai Republic |
| Km_79 | M9a1b2 | 223 234 362 | 73 150 152 153 188 263 309.1C 315.1C | Kalmyk | Russia, Kalmyk Republic |
| Kor_10 | M9a4b | 223 234 362 | 73 153 263 309.1C 315.1C | Korean | South Korea, Seoul |
| Khm_36 | N9a1 | 111 129 223 257CA 261 | 73 150 200 263 315.1C | Khamnigan | Russia, South Siberia, Buryat Republic |
| Kor_87 | N9a1 | 111 129 223 257CA 261 | 73 150 263 309.1C 315.1C | Korean | South Korea, Seoul |
| Bt_122 | N9a2* | 172 223 257CA 261 278 | 73 150 263 309.1C 309.2C 315.1C | Barghut | China, Inner Mongolia, Hulun Buir Aimak |
| Tat_ 411G | N9a2a2 | 172 223 257CA 261 | 73 150 263 309.1C 315.1C | Tatar | Russia: Volga-Ural Region |
| Bt_81 | N9a2a3 | 172 223 257CA 261 305AT (497) | 73 150 263 309.1C 315.1C | Barghut | China, Inner Mongolia, Hulun Buir Aimak |
| Br_433 | N9a3 | 129 223 257CA 261 | 73 150 263 309.1C 315.1C | Buryat | Russia, South Siberia, Buryat Republic |
| Kor_92 | N9a3 | 129 223 257CA 261 278 | 73 150 195 263 309.1C 309.2C 315.1C | Korean | South Korea, Seoul |
| Rus_BGII-19 | N9a3a | 129 223 257CA 261 | 73 150 263 309.1C 315.1C | Russian | Eastern Europe, Russia, Belgorod region |
| Cz_V-44 | N9a3a | 129 223 257CA 261 | 73 150 263 309.1C 315.1C | Czech | Eastern Europe, Czech Republic, West Bohemia |
| Br_623 | N9a8 | 223 257CA 261 | 73 150 263 315.1C | Buryat | Russia, South Siberia, Buryat Republic |
| Bt_120 | R9c1a2 | 051 304 335 362 | 44.1C 73 146 150 151 263 309.1C 315.1C | Barghut | China, Inner Mongolia, Hulun Buir Aimak |
| Alt_158 | R11b1 | 092 095 189 311 | 73 185 189 263 309.1C 315.1C | Altaian-Kizhi | Russia, South Siberia, Altai Republic |
